# Cuproptosis patterns in papillary renal cell carcinoma are characterized by distinct tumor microenvironment infiltration landscapes

**DOI:** 10.3389/fmolb.2022.910928

**Published:** 2022-10-05

**Authors:** Chiyu Zhang, Ruizhen Huang, Xiaoqing Xi

**Affiliations:** Department of Urology, The Second Affiliated Hospital of Nanchang University, Nanchang, China

**Keywords:** cuproptosis, papillary renal cell carcinoma, tumor microenvironment, drug sensitivity, prognostic model

## Abstract

Cuproptosis is a novel kind of programmed cell death that has been linked to tumor development, prognosis, and responsiveness to therapy. Nevertheless, the precise function of cuproptosis-related genes (CRGs) in the tumor microenvironment (TME) remains unknown. We characterized the genetic and transcriptional changes of CRGs in papillary renal cell carcinoma (PRCC) samples and analyzed the expression patterns in two separate cohorts. We observed that two unique cuproptosis-related subgroups and three separate gene subgroups were connected with clinicopathological, prognostic, and TME features of patients. Then, a risk score for predicting overall survival (OS) was created and validated in patients with PRCC. To make the risk score more clinically useful, we created a nomogram that was very accurate. A lower risk score, which was associated with higher tumor mutation burden, and immune activity, suggested a better prognosis for OS. Additionally, the risk score was shown to be substantially linked with the drug’s susceptibility to chemotherapeutic agents. Our extensive research of CRGs in PRCC identified possible roles for them in the TME, clinicopathological features, and overall survival. These findings may help advance our knowledge of CRGs in PRCC and pave the way for improved prognosis and the creation of more effective immunotherapy therapies.

## Introduction

Renal cell carcinoma (RCC) is the most common kind of renal tumor, accounting for up to 80% of cases; papillary renal cell carcinoma (PRCC) is the second most prevalent type of RCC, accounting for around one-fifth of all instances (Mendhiratta et al., 2021; Rysz et al., 2021). Patients with localized PRCC have a reported 5-year overall survival rate of 70%, whereas patients with advanced PRCC do not have any feasible therapy choices at this time (Akhtar et al., 2019; Steward et al., 2021; Chan et al., 2022). Currently, an increasing number of clinical investigations have been conducted on individuals with clear cell RCC and have identified many efficacious treatment targets, including VEGFR and mTOR (Erlmeier et al., 2022; Labaki et al., 2022). Nevertheless, these strategies were less effective in PRCC patients, which may be due to the fact that PRCC carcinogenesis involves distinct genetic alterations and molecular pathways from clear cell RCC tumorigenesis (Paner et al., 2022). As a result, new precise biomarkers and effective treatment techniques for PRCC are required.

Copper (Cu) is a necessary cofactor for all species, but it becomes hazardous when quantities reach a homeostatic threshold (Ruiz et al., 2021). Nevertheless, the mechanism by which excess copper causes cell death is uncertain. In human cells, Tsvetkov et al. demonstrated that Cu-dependent, controlled cell death is unique from other known cell death processes and requires mitochondrial respiration (Tsvetkov et al., 2022). It established that copper-dependent mortality occurs as a result of copper’s direct binding to lipoylated tricarboxylic acid (TCA) cycle components. This leads to the aggregation of lipoylated proteins and the loss of iron-sulfur cluster proteins, which causes a lot of stress on the body and eventually cell death. They demonstrated that copper toxicity is unique from all other known processes of controlled cell death, such as apoptosis, ferroptosis, pyroptosis, and necroptosis (Tsvetkov et al., 2022). As a result, they suggest the name “cuprotosis” for this hitherto uncharacterized cell death process. Despite this, a number of associations between illness and Cu have been discovered. Cu levels have been shown to be greater in several cancers than in normal tissues in various investigations (Stepien et al., 2017; Aubert et al., 2020; Saleh et al., 2020; Michniewicz et al., 2021). Cu deposition has been linked to increased proliferation and growth, as well as angiogenesis and metastasis (Oliveri, 2022). Cu dyshomeostasis is clearly important in cancer, although scientists disagree over whether it is a cause or a result of carcinogenesis.

The tumor microenvironment (TME) is a complex and ever-changing milieu that mostly consists of stromal cells and immune cells (Hinshaw and Shevde, 2019). Cancer develops and progresses in conjunction with changes in the surrounding stroma (Wu and Dai, 2017). Through the production of different cytokines, chemokines, and other substances, cancer cells may effectively design their microenvironment (Vitale et al., 2019). This results in the surrounding cells’ being reprogrammed, allowing them to play an important part in the proliferation of cancer cells (Kochetkova and Samuel, 2022). Immune cells are essential components of the tumoral microenvironment and are required for this process to occur. The growing body of evidence indicates that when innate and adaptive immune cells interact in the TME, they promote tumor development (Hedrick and Malanchi, 2022). The interaction of cancer cells and their proximal immune cells eventually leads to an environment conducive to tumor development and spread (Burrello and de Visser, 2022). Trying to figure out how this interaction works could lead to better medicines that can affect many parts of the TME at the same time, which could lead to better patient treatment results (Bader et al., 2020).

We conducted a detailed analysis of cuproptosis-related genes and their relationship to the progression, prognosis, and immune response of PRCC in detail. We identified distinct cuproptosis patterns in PRCC using The Cancer Genome Atlas (TCGA) and Gene Expression Omnibus (GEO) datasets and assessed the clinical features, prognostic significance, and immune infiltration degree of the resultant cuproptosis clusters. Additionally, we created a cuproptosis score that accurately predicted patients with PRCC’s prognosis and therapy responsiveness. These results may aid in the development of successful immunotherapies for PRCC.

## Materials and methods

### Obtaining and processing raw data

The transcriptional mRNA sequences (fragments per kilobase of transcript per million, FPKM) and clinicopathological data for PRCC samples were obtained from TCGA and GEO databases. For the following analyses, data from the Cancer Genome Atlas’s kidney renal papillary cell carcinoma (TCGA-KIRP) dataset and the Gene Expression Omnibus Series 2748 (GSE 2748) dataset were collected. We used the raw “CELL” files to modify the backdrop and normalize the quantiles. The FPKM values of TCGA-KIRP were converted to transcripts per kilobase million (TPM) and were thought to be equivalent to those from microarray data (Zhao et al., 2021). The batch effects from nonbiological technical biases in the two datasets were removed using the ComBat algorithm from the “SVA” package (Leek et al., 2012). The TCGA database was used to get data on somatic mutations and copy number variation (CNV).

### Unsupervised clustering study of cuproptosis-related genes

Thirteen cuproptosis-related genes (CRGs) were extracted from prior studies, including FDX1, LIPT1, LIAS, DLD, DBT, GCSH, DLST, DLAT, PDHA1, PDHB, SLC31A1, ATP7A, and ATP7B(Cobine et al., 2021; Tsvetkov et al., 2022). To categorize individuals into discrete molecular subgroups based on cuproptosis-related gene (CRG) expression, the R package “ConsensusClusterPlus” was used for consensus unsupervised clustering analysis (Wilkerson and Hayes, 2010). This grouping was carried out using the following standards: To begin, the cumulative distribution function (CDF) curve steadily and gently expanded in magnitude. Secondly, there were no small sample sizes in any of the categories. Finally, following clustering, the correlation inside groups grows and the correlation between groups diminishes. The research was conducted a total of 1000 times to confirm its accuracy as a clustering tool. We investigated the connections between genetic subclusters and clinicopathological features to determine the clinical utility of the two subgroups determined by consensus clustering. Additionally, we utilized Kaplan–Meier curves generated by the R tools “survival” and “survminer” to assess differences in overall survival (OS) among distinct subclusters (Lv et al., 2021).

### Correlations between subclusters and the tumor microenvironment

To get a better understanding of the biological roles within distinct CRG subclusters, we utilized the “GSVA” R package to conduct gene set variation analysis (GSVA) analyses on each CRG subcluster (Hänzelmann et al., 2013). The immunological and stromal scores of each patient were calculated using the ESTIMATE method. Additionally, the CIBERSORT method was used to compute the percentages of 23 human immune cell types in each PRCC sample (Chen et al., 2018; Zhang et al., 2021). Additionally, we estimated the levels of immune cell infiltration in the tumor microenvironment using a single-sample gene set enrichment analysis (ssGSEA) approach (Mao et al., 2022).

### Identification of DEGs

The R tool “limma” was used to compare differentially expressed genes (DEGs) amongst CRG subclusters (Ritchie et al., 2015). Then, Gene Ontology (GO) enrichment analysis was used to assess biological functions, and Kyoto Encyclopedia of Genes and Genomes (KEGG) enrichment analysis was used to evaluate regulatory pathways (Gene Ontology Consortium, 2015; Kanehisa et al., 2017).

### The calculation of risk scores

A scoring system was developed to measure the cuproptosis gene alteration patterns in each PRCC patient. To begin, DEGs were screened across several CRG subclusters, with crossing DEGs maintained for further research. To assess the aforementioned intersecting DEGs and to filter for genes linked with PRCC prognosis, we employed univariate Cox regression techniques. Following that, we employed an unsupervised clustering technique to divide PRCC patients into distinct subclusters for a full systematic analysis based on prognosis-related genes. Additionally, we used Principal Component Analysis (PCA) to identify genes strongly linked with prognosis in order to develop cuproptosis-relevant gene signatures. The PCA approach enabled the scores to be concentrated on highly associated gene modules and downscaled for modules with modest contributions or correlations. Finally, we established cuproptosis scores for each PRCC patient using a mechanism identical to that used to rate gene expression. The following equation was used to get the risk score: Risk score = Σ (Expi * Coefi) (Coefi denotes the risk coefficient and Expi the gene expression). 

### Developing and validating a nomogram-based scoring system

Based on the conclusion of the independent prognosis study, we utilized the clinical parameters and risk score to build a prediction nomogram using the “rms” software. Each variable was assigned a score in the nomogram scoring method, and the overall score was calculated by summing the scores for all variables for every subject. The nomogram was evaluated using time-dependent receiver operating characteristic (ROC) curves for survivals (Obuchowski and Bullen, 2018). The nomogram’s calibration plots were utilized to illustrate the prognostic validity between expected survival events and practically actual outcomes.

### Analyses of mutations and drug susceptibility

The “maftools” R package was used to construct the mutation annotation format (MAF) from the TCGA in order to compare the somatic mutations of PRCC patients in two subgroups (Mayakonda et al., 2018; Ferrer-Bonsoms et al., 2021). The tumor mutation burden (TMB) score for each patient with PRCC in the two groups was also computed. To examine whether there were any differences in the therapeutic effects of chemotherapeutic medications in the two subgroups, we utilized the “pRRophetic” package to determine the semi-inhibitory concentration (IC50) values of chemotherapy agents routinely used to treat PRCC (Geeleher et al., 2014; Wang et al., 2021).

### Statistical analysis

The Wilcoxon rank-sum test was used to make comparisons between two groups. The Kruskal-Wallis test was used for comparisons of three or more groups. Survival studies including risk scores were carried out using the Kaplan-Meier technique. The log-rank test was used to examine the difference in survival statistics. The function “surv-cutpoint” was used to determine the best cut-off for the cohort in order to categorize patients into high and low-risk score subgroups. The Univariate and multivariate Cox regressions were used to assess the prognostic significance of the risk score. R software version 4.2.0 was used for all data analysis. A statistically significant *p*-value of 0.05 was defined.

## Results

### Cuproptosis-related genes genetic variation landscape in papillary renal cell carcinoma

**FIGURE 1:**
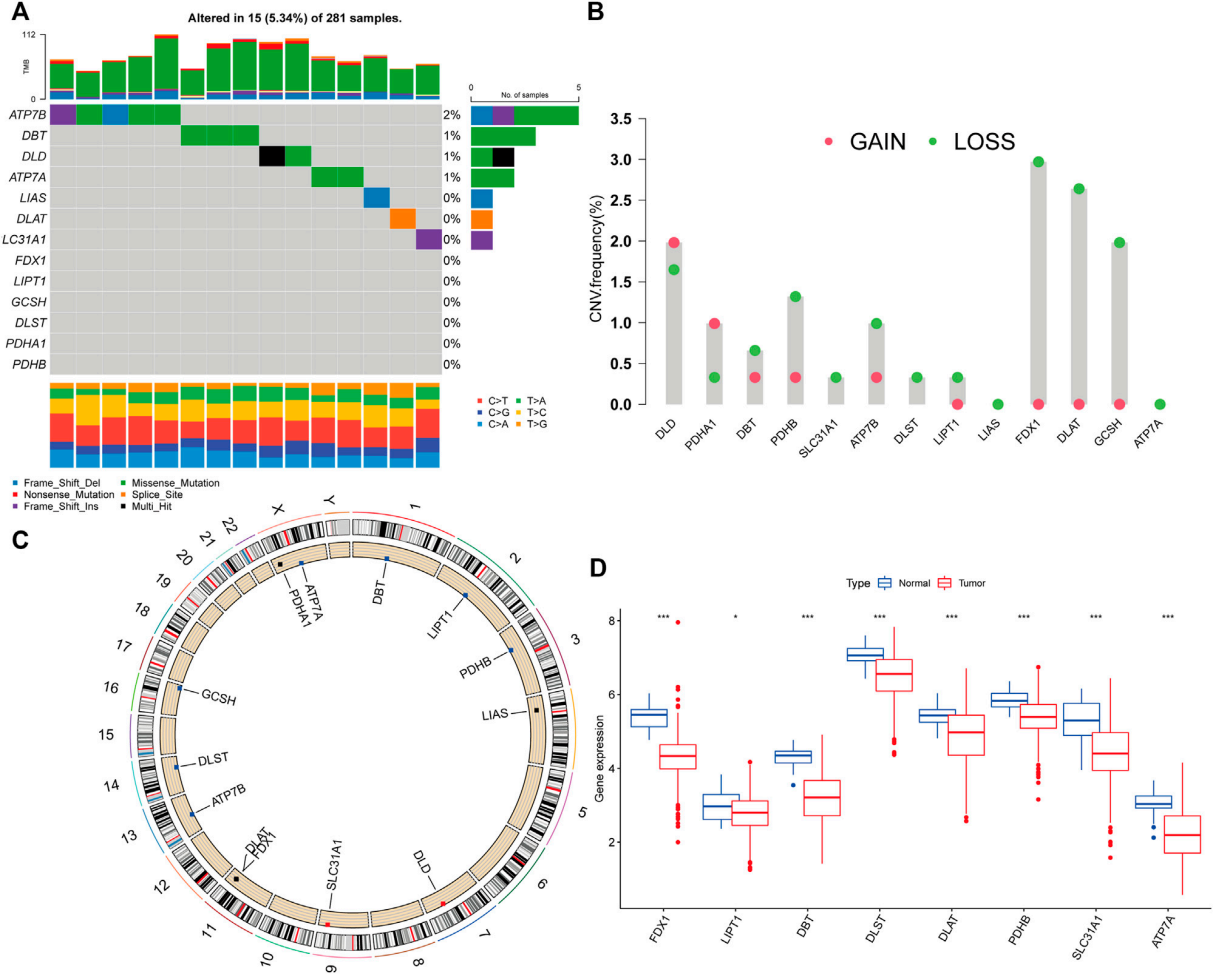
Cuproptosis-related genes (CRGs) genetic variation landscape in papillary renal cell carcinoma (PRCC). **(A)**The frequency of CRGs mutations in 281 patients with PRCC. On the right, the number indicated the mutation frequency of the CRGs. **(B)**The CRGs’ copy number variation (CNV) frequency. The column indicated the frequency of variation. Red: gain frequency. Green: loss frequency. **(C)**The chromosomal location of the CRGs with a CNV. **(D)** Analysis of the mRNA expression of CRGs in normal and PRCC tissues. Blue: normal renal tissue. Red: tumor tissue. (*p < 0.05; ***p < 0.001).

The TCGA dataset was used to investigate the landscape of genetic variants in 13 CRGs in PRCC, including somatic mutation and CNV. Genetic variations in CRGs were found in 15 out of the 281 samples (5.34 percent), with the majority of the variants being missense mutations (Figure 1A). The most often mutated gene was ATP7B, followed by DBT, DLD, and ATP7A, but PDHB, PDHA1, DLST, GCSH, LIPT1, FDX1, SLC31A1, DLAT, and LIAS did not mutate in PRCC samples. Following that, we examined somatic CNVs in these CRGs and determined that they were widespread in 11 CRGs (Figure 1B). DLD and PDHA1 exhibited increased CNV frequency, but DBT, PDHB, SLC31A1, ATP7B, DLST, LIPT1, FDX1, DLAT, GCSH, and GCSH all had decreased CNV frequency. Each chromosome in Figure 1C has been colored in to illustrate where each CRG has a copy number variation. We also analyzed the transcriptional level of CRGs in PRCC and normal tissues, and discovered that the transcriptional levels of most CRGs were positively linked with the incidence of CNV. CNV-deficient CRGs, including FDX1, DLAT, DLST, PDHB, SLC31A1, ATP7A, and DBT, were expressed at lower levels in PRCC samples than in renal samples, suggesting that CNVs may regulate CRG mRNA expression (Figure 1D). As a result, the genomic and transcriptome landscape in CRGs is critical for controlling the onset and development of PRCC.

### Identification of cuproptosis subclusters in papillary renal cell carcinoma

**FIGURE 2:**
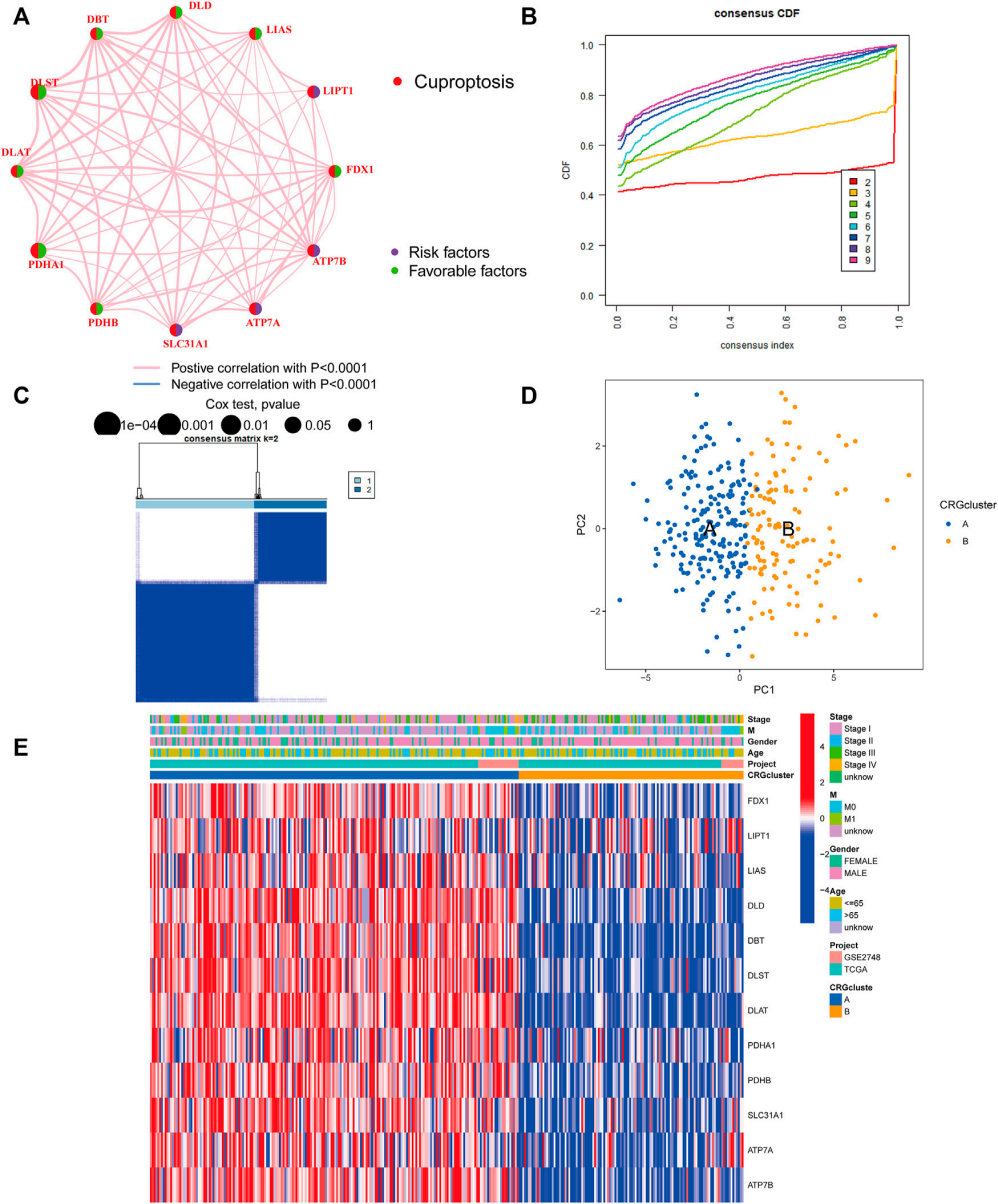
Identification of cuproptosis subclusters in PRCC. **(A)** Interactions among CRGs in PRCC. Greater PRCC predictive influence is shown by larger circles. The protective factor is represented by green, and the risk prognostic factor by the dark blue within the circle. **(B)** Consensus clustering cumulative distribution function (CDF) curve when K = 2–9. **(C)** The consensus clustering matrix for CRG modification patterns. At K = 2, the samples are partitioned with reasonable stability. **(D)** Principal component analysis (PCA) of two clusters. Blue indicates CRG cluster A, whereas orange represents CRG cluster B. **(E)** The heatmap depicts the expression of CRGs and clinicopathologic characteristics in different subclusters. Red denotes high CRG expression and blue, low CRG expression.

The TCGA-PRCC and GSE2748 were combined to create a meta-cohort. Each dataset comprised comprehensive clinicopathological information and survival data. The network depicted a full panorama of the CRGs’ expression levels, correlations, and prognostic significance in PRCC patients (Figure 2A). These findings suggest that cross-talk between CRGs is crucial for the development of cuproptosis patterns in individuals. To understand more about the CRGs’ expression properties in PRCC, we used a consensus clustering approach to identify patients with PRCC based on their transcriptional levels (Figures 2B,C). According to our results, the optimal option for subdividing the whole cohort into subclusters A (*n* = 199) and B (*n* = 122) is k = 2. At K = 2, the samples are partitioned with reasonable stability. The cuproptosis transcriptional patterns of the two subclusters differed significantly according to PCA analysis (Figure 2D). Furthermore, evaluating the clinicopathological characteristics of different CRG subclusters revealed significant differences in CRG transcription and pathological stage. Additionally, we detected substantial changes in CRG expression across various cuproptosis patterns, with all CRGs being downregulated in CRG cluster B and upregulated in CRG cluster A (Figure 2E).

**FIGURE 3:**
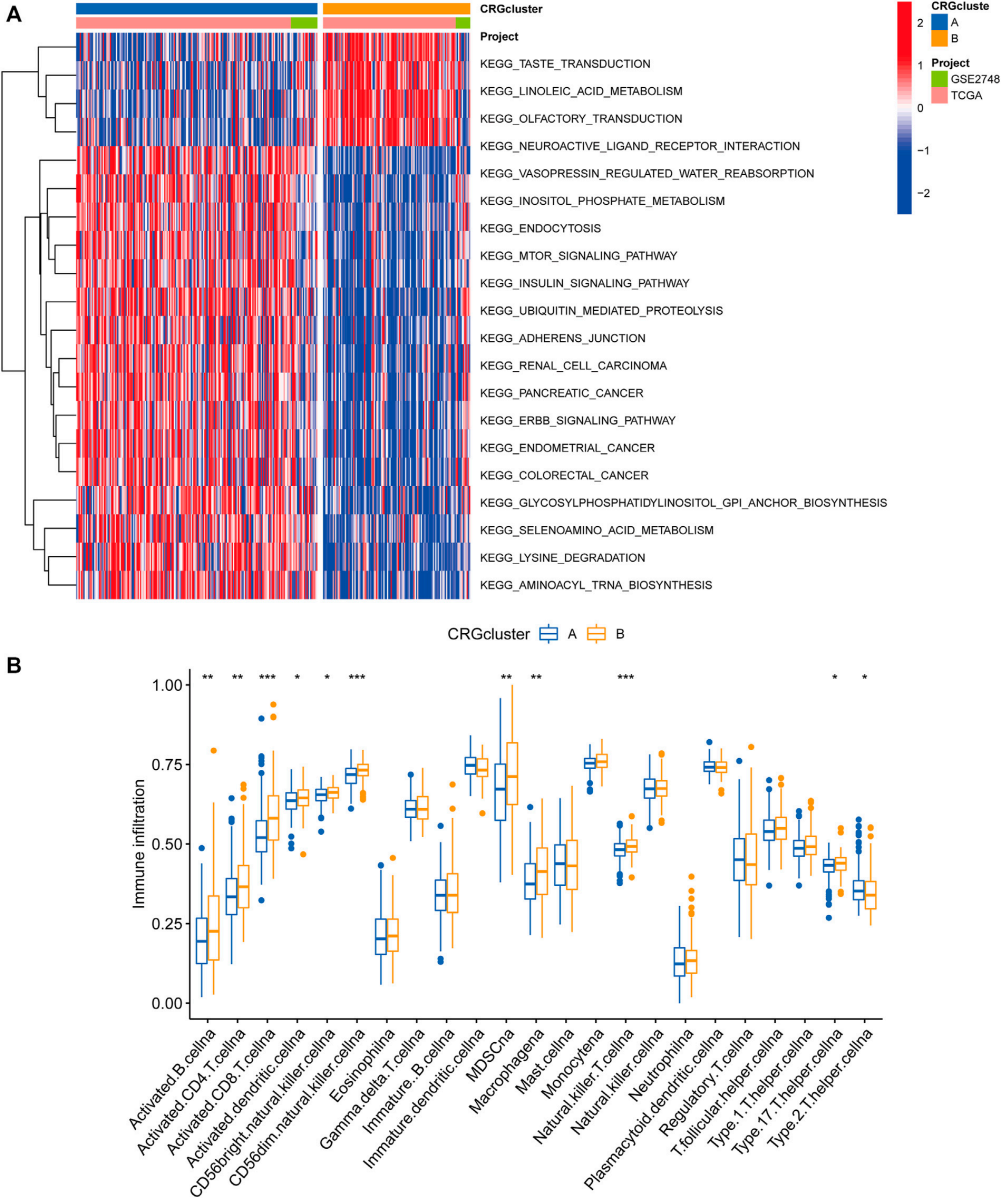
Correlations between the microenvironment of tumor immune cells and two PRCC subclusters. **(A)** Gene set variation analysis (GSVA) of biological pathways divided into two separate subclusters, with red denoting active pathways and blue denoting inhibited pathways, respectively. **(B)** The degrees of tumor microenvironment immune cell infiltration between the two CRG clusters. Blue symbolizes cluster A, whereas orange represents cluster B. The median value is indicated by the thick line, and the interquartile range by the bottom and top of the box. The dispersed dots signify anomalies. (*p < 0.05; **p < 0.01; ***p < 0.001).

Following that, we examined the molecular biological characteristics associated with the two cuproptosis clusters. The GSVA analysis revealed that CRG cluster A was significantly enriched in tumor-associated pathways, including the renal cell carcinoma pathway, pancreatic cancer pathway, endometrial cancer pathway, and colorectal cancer pathway (Figure 3A). Using the CIBERSORT method, we examined the correlations between the two subclusters and 23 human immune cell subtypes of each PRCC sample to explore the involvement of CRGs in the TME of PRCC. According to our findings, the infiltration of most immune cells differed significantly between the two subclusters (Figure 3B). Subcluster B had significantly more activated B cells, CD4 T cells, CD8 T cells, activated dendritic cells, CD56bright natural killer cells, CD56dim natural killer cells, MDSC, Macrophage, and natural killer T cells than subcluster A.

### Gene classification based on differentially expressed genes

**FIGURE 4:**
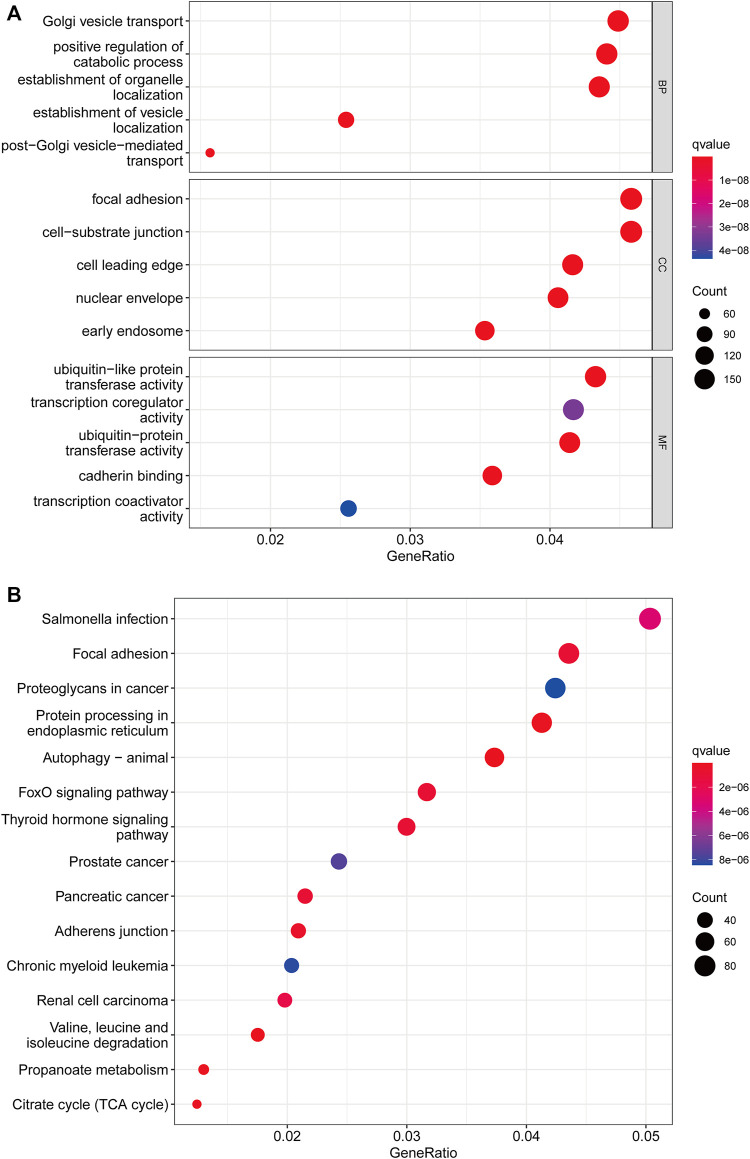
Functional enrichment analysis. **(A)** Bubble plot for Gene Ontology (GO) function enrichment analysis. BP: biological processes; CC: cellular components; MF: molecular function. **(B)** Bubble plot for Kyoto Encyclopedia of Genes and Genomes (KEGG) pathway enrichment analysis. The y-axis shows pathway terms, whereas the x-axis denotes gene ratio. The size of each circle represents the number of genes. The hue of the circles symbolizes various q values.

We used the “limma” R package to search for 3977 cuproptosis subcluster-related DEGs, identified as CRG signature genes, to better understand the probable biological roles across distinct CRG clusters (Figure 4A). The “ClusterProfile” R package was then used to conduct GO functional and KEGG pathway enrichment studies to annotate and show DEGs’ biological functions. DEGs were found to be significantly overrepresented in cellular metabolism-associated pathways. In biological processes, DEGs were enriched in Golgi vesicle transport, establishment of organelle localization, and positive regulation of catabolic process. In cellular components, DEGs were highly abundant in focal adhesion, cell−substrate junction, and cell leading edge. DEGs were considerably enriched in ubiquitin−like protein transferase activity, transcription coregulator activity, and ubiquitin−protein transferase activity throughout molecular function processes (Figure 4B). DEGs were also highly enriched in tumor-associated pathways in KEGG analyses: proteoglycans in cancer, prostate cancer, pancreatic cancer, chronic myeloid leukemia and renal cell carcinoma (Figure 4B).

**FIGURE 5:**
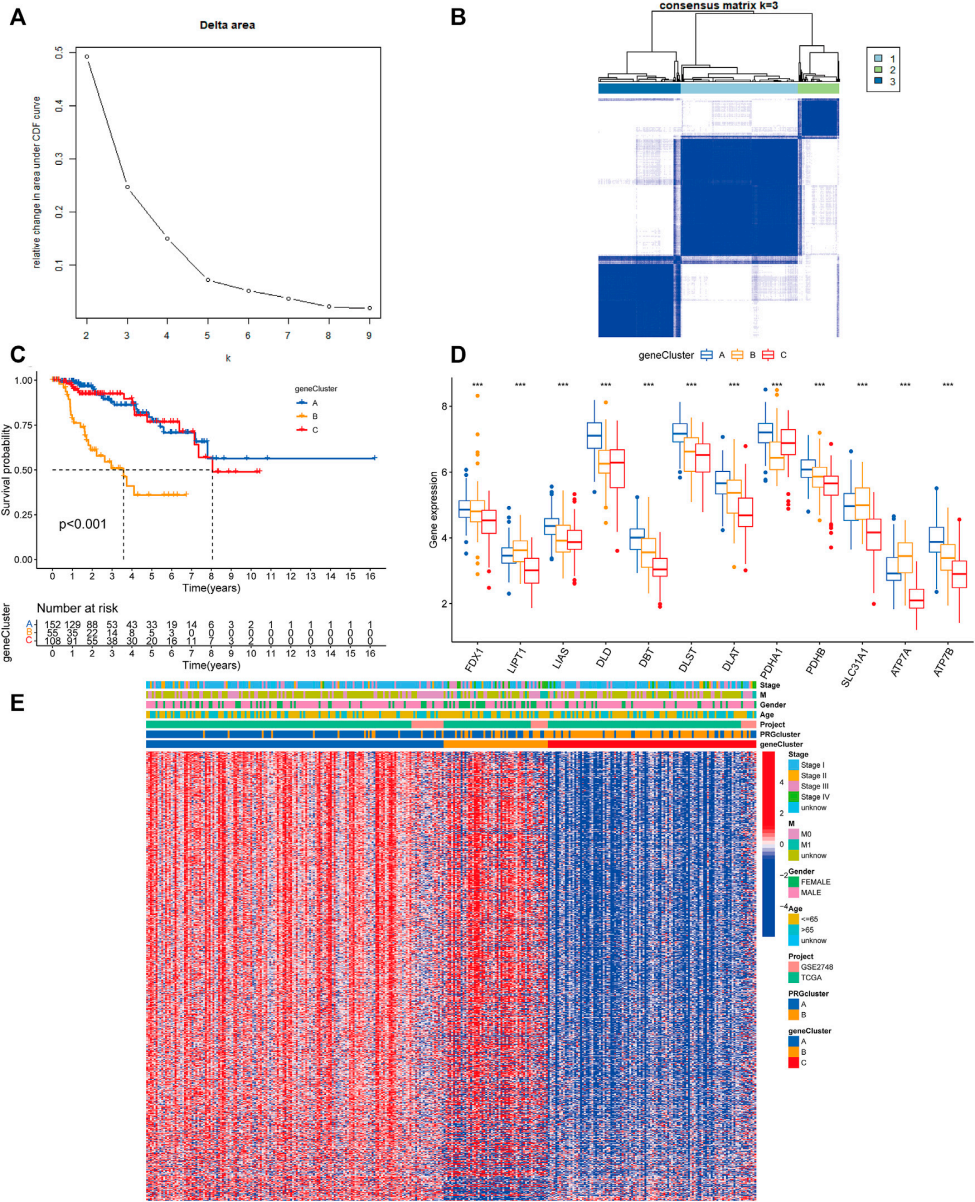
Gene classification based on differentially expressed genes. **(A)** For K = 2–9, the relative change in the area under the CDF curve. **(B)** Heatmap of the consensus matrix identifying two clusters (k = 3) and the region of their correlation. At K = 3, the samples are partitioned with reasonable stability. **(C)** Kaplan–Meier curves for the overall survival of the gene subclusters. Blue indicates gene cluster A, orange cluster B, and red cluster C. Log-rank *p* < 0.001, suggesting a substantial difference among the three gene clusters in terms of overall survival. Cluster B’s overall survival was much worse than clusters A and C’s **(D)** CRG expression differences between gene subclusters. An interquartile range of the data was indicated by the upper and lower ends of the boxes. The boxes’ lines indicated the median value. (one-way ANOVA test: ***p < 0.001). **(E)** A heat map of the clinical-pathologic correlations between the two gene clusters. Alternate annotations are provided for age, gender, pathologic staging, and gene clusters. Blue denotes low gene expression whereas red denotes high gene expression.

Following that, we used univariate Cox regression to assess the prognostic value of 3977 subcluster-related genes and identified 739 genes linked with OS time for further analysis (*p* < 0.05). We conducted an unsupervised cluster analysis on the 739 DEGs associated with prognosis to group PRCC patients into three distinct gene subclusters: gene subcluster A, gene subcluster B, and gene subcluster C (Figures 5A,B). At K = 3, the samples are partitioned with reasonable stability. Patients with gene subcluster B had the poorest overall survival, while patients in gene subcluster A had the best OS (*p* < 0.001, Figure 5C). CRG expression differed significantly amongst the three cuproptosis gene subclusters, as predicted based on the cuproptosis patterns (Figure 5D). This indicated that greater CRG expression may be associated with a better prognosis for individuals with PRCC. Additionally, the heatmap of gene expression indicated that these differentially expressed genes associated with prognosis were strongly expressed in gene cluster B (Figure 5E).

### Developing the prognostic risk score

**FIGURE 6:**
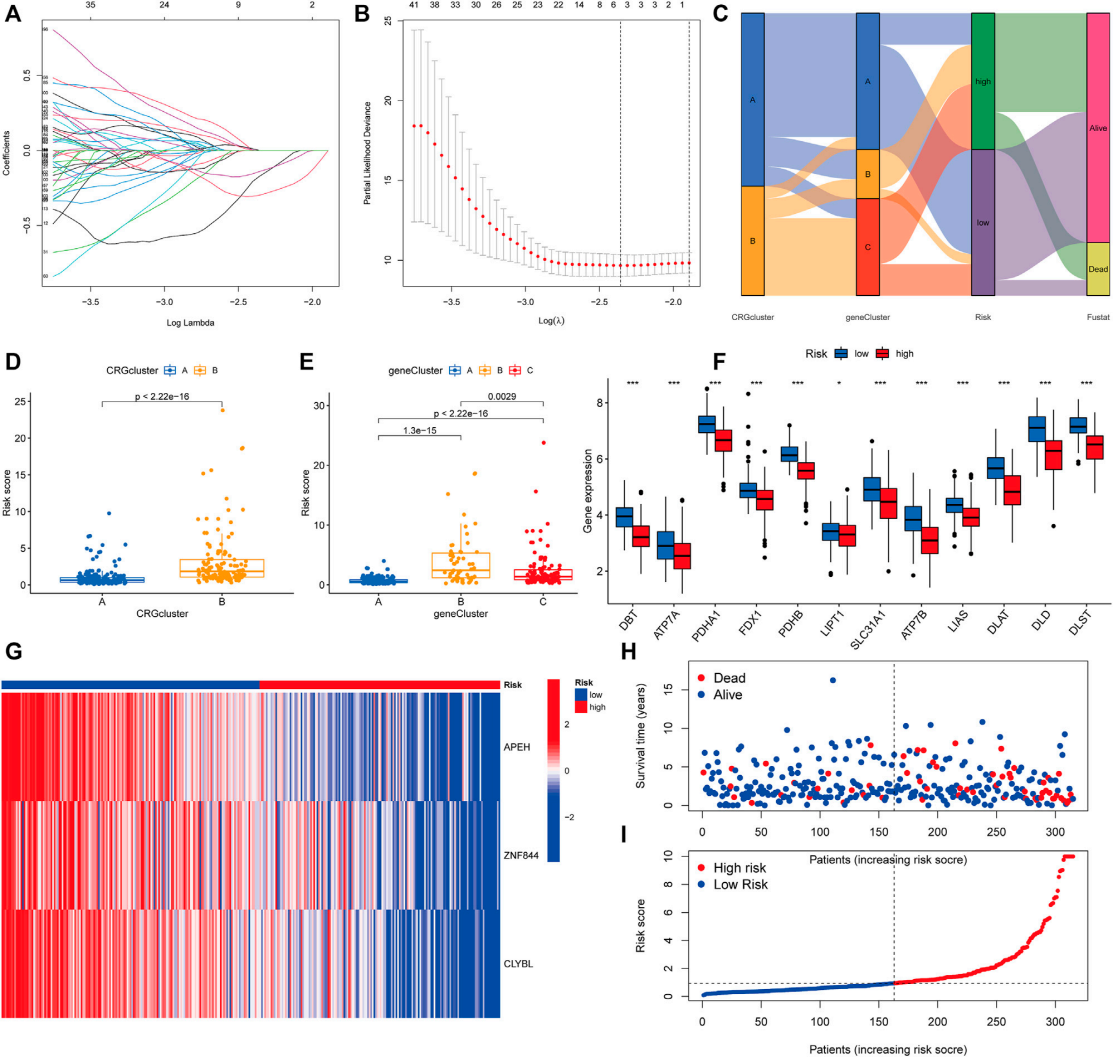
Developing the prognostic risk score. **(A,B)** Least absolute shrinkage and selection operator (LASSO) regression and partial likelihood deviance on prognostic genes. **(C)** Distributions of various CRG clusters, gene clusters, risk scores, and survival outcomes are shown in alluvial plots. **(D)** Differences in risk scores between two CRG clusters. The Wilcoxon rank-sum test revealed that the differences between the two clusters were statistically significant (*p* < 0.001). **(E)** Differences in risk scores among three gene clusters. The Kruskal-Wallis test (*p* < 0.001) was used to assess the differences between the three gene clusters. **(F)** Histogram of mRNA expression of CRG between high-risk and low-risk score groups. **(G)** Heatmap of three significant genes across different risk scores. **(H,I)** Distribution of risk scores and patient survival status as shown by dot and scatter plot.

To begin, we used the R package “caret” to randomly assign patients to one of two subgroups: training (*n* = 158) or testing (*n* = 157). The optimum predictive signature for 739 cuproptosis subcluster-related DEGs was further refined using least absolute shrinkage and selection operator (LASSO) regression and multivariate Cox regression analysis. Following LASSO regression analysis, the least partial likelihood deviance revealed that 5 OS-related genes remained (Figures 6A,B). Then, using the Akaike information criterion (AIC) value, we did multivariate Cox regression analysis on 5 OS-related genes to yield three significant genes (APEH, ZNF844, and CLYBL). The DEGs linked with the subclusters were used to generate the risk score. The distribution of patients into two CRG subclusters, three gene subclusters, and two risk score subgroups is shown in Figure 6C. More crucially, CRG cluster B showed a considerably higher risk score than CRG cluster A (Figure 6D). Between gene subclusters, we discovered a substantial variation in risk score. The risk score for gene subcluster A was the lowest, while that for gene subcluster B was the highest, suggesting that a low-risk score is likely to be associated with immunological activation-related characteristics, whilst a high-risk score is likely to be associated with stromal activation-related characteristics (Figure 6E). It was discovered that all CRGs were considerably overexpressed in the low-risk subgroup (Figure 6F). Similarly, as seen in the heatmap, the three genes included in the score were significantly expressed in the low-risk subgroup (Figure 6G). The distribution plot demonstrated that as risk scores climbed, survival times were reduced and recurrence rates increased (Figures 6H,I).

### The construction of a nomogram for survival prediction

**FIGURE 7:**
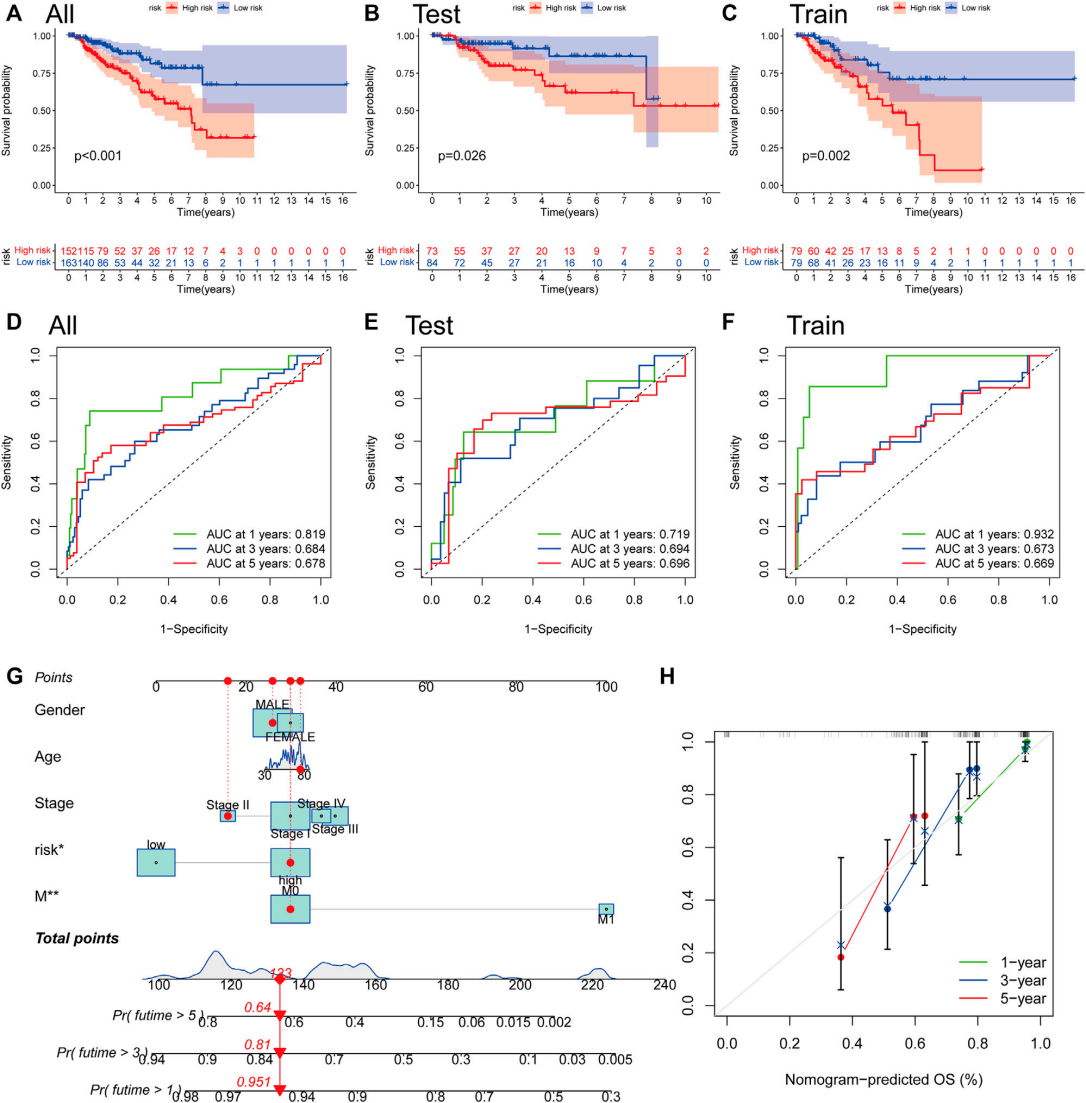
The construction of a nomogram for survival prediction. **(A–C)** Overall Survival analysis using the Kaplan–Meier method in all, training, and testing sets. **(D–F)** Time-dependent receiver operating characteristic (ROC) curves to estimate the sensitivity and specificity of survival in all, training, and testing sets based on the risk score. **(G)** Nomogram for predicting overall survival in patients with PRCC. **(H)** The nomogram’s calibration curves for predicting overall survival. *p < 0.05, **p < 0.01.

We computed risk scores across testing and training sets to confirm the risk score’s predictive performance. According to the methodology used for the whole set, the patients were likewise divided into two risk categories. Survivability studies showed that patients in the lower-than-normal risk category had a considerably better prognosis (Figures 7A–C). The AUC values for the risk score at 1, 3, and 5 years were 0.819, 0.684, and 0.678, respectively, in the all set (Figure 7D). There were 0.719, 0.694, and 0.696 AUC values for the risk score at 1, 3, and 5 years in the testing set (Figure 7E). Similarly, the training group’s AUC values are 0.932, 0.673, and 0.669, correspondingly (Figure 7F). One, three, and five-year prognostic efficiency AUC values for the risk score were demonstrated to be quite high, indicating that the risk score had a remarkable ability to predict the life expectancy of people with PRCC. Because the risk score is difficult to apply in practice, we created a nomogram that combines the risk score with clinicopathological variables to estimate patient survival time. As predictors of the nomogram, we used the risk score, gender, age, tumor metastasis, and cancer stage as variables to consider (Figure 7G). In particular, the calibration plots revealed that the nomograms we developed functioned in a manner comparable to the ideal model, particularly when it came to the one-year survival period (Figure 7H).

### A comparative analysis of the tumor microenvironment in various risk categories

**FIGURE 8:**
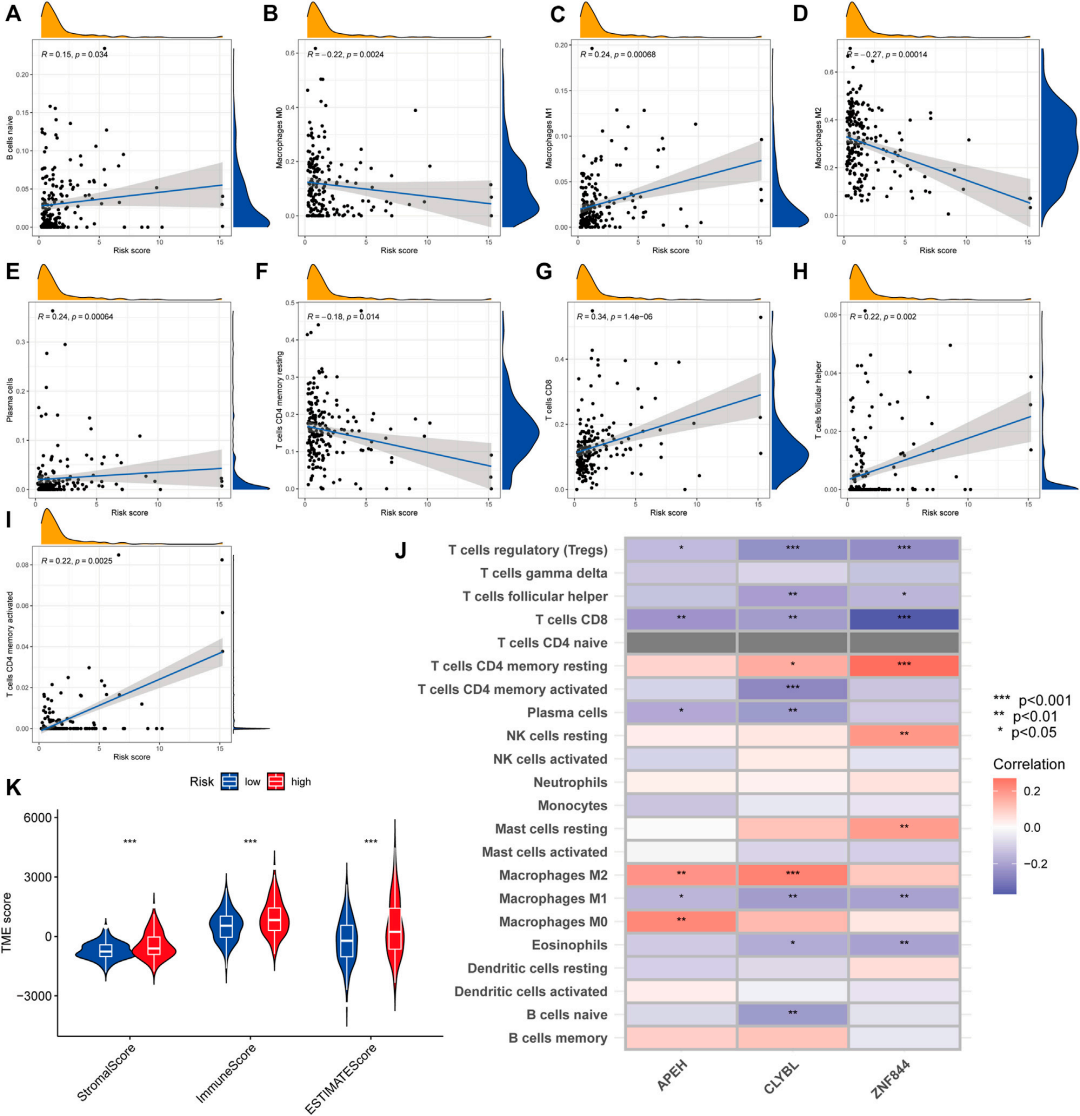
Comparative analysis of tumor microenvironment in various risk categories. **(A–I)** Relationship between the risk score and the kind of immune cells. R represents the correlation coefficient, and when it is positive, it means that immune cell infiltration is positively correlated with the risk score, and *vice versa*. **(J)** Relationship between immune cell abundance and three genes included in the suggested model. Red represents a positive correlation between immune cell infiltration and gene expression, and blue represents a negative correlation. The darker the color, the greater the correlation. **(K)** Relationship between risk score and both immune and stromal scores. A greater risk score was shown to be substantially associated with a higher immunological score, stromal score, and ESTIMATE score.

We employed the CIBERSORT technique to assess the connection between the risk score and immune cell abundance. As demonstrated in the scatter graphs, the risk score was positively linked with B cells, M1 Macrophages, Plasma cells, CD8 T cells, follicular helper T cells, and CD4 memory activated T cells but negatively associated with M0 Macrophages, M2 Macrophages, and CD4 memory resting T cells (Figures 8A–I). We identified a substantial association between the majority of immune cells and three genes, including APEH and M0 Macrophages, CLYBL and M2 Macrophages, and ZNF844 and CD4 memory resting T cells (Figure 8J). A greater risk score was also shown to be substantially associated with a higher immunological score, stromal score, and ESTIMATE score (Figure 8K).

### Analyzing genetic mutations and drug susceptibility

**FIGURE 9:**
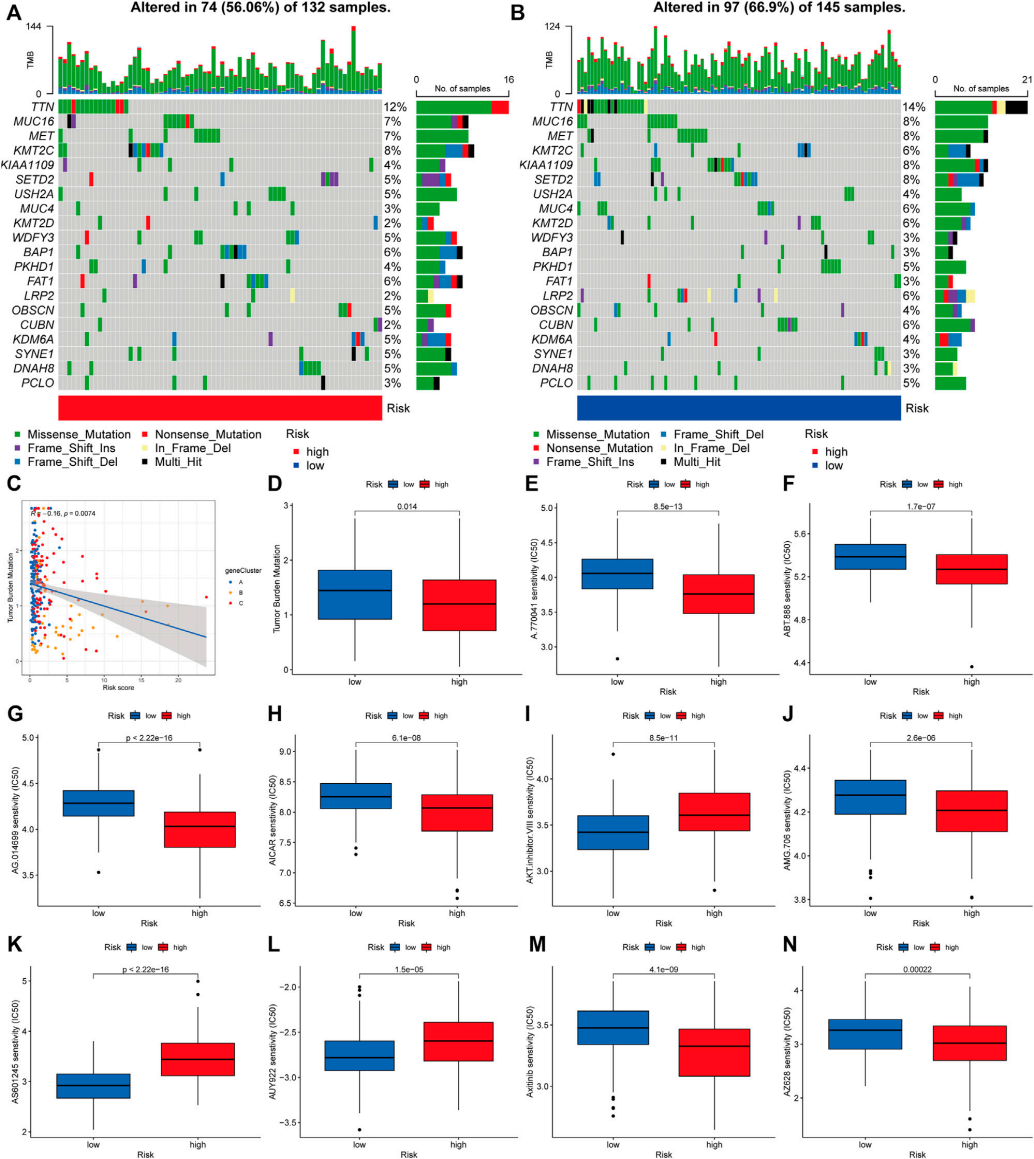
Analyzing genetic mutations and drug susceptibility. **(A,B)** The waterfall plot depicting the somatic mutation characteristics associated with various risk scores. The numbers on the graph show the frequency of mutation. The fraction of mutation types is shown by the box on the right. **(C)** Spearman correlation study of risk score and tumor mutational burden. R represents the correlation coefficient, and when it is negative, it means that the tumor burden mutation is negatively correlated with the risk score. **(D)** Tumor mutational burden in several risk score categories. **(E–N)** The relationship between risk score and drug sensitivity. Red represents the high-risk group and blue represents the low-risk group. The lower the half maximal inhibitory concentration (IC50) value, the more sensitive the group of patients to the drug.

According to accumulating research, due to their large amounts of mutant antigens, people with a high TMB may react better to immunotherapy than those with a low TMB. As a further step, we compared the somatic mutation distribution across two risk score subgroups (Figures 9A,B). The Spearman correlation analysis showed that the risk score and tumor mutational burden were linked in a negative way (R = -0.16, *p* = 0.0074; Figure 9C). Our examination of the mutation datasets revealed that the higher-risk category had a lower TMB than the lower-risk category, suggesting that the lower-risk category may benefit from immunotherapy (Figure 9D). The top twenty mutated genes were similar in both groups, but the majority of genes in the lower-risk subgroup had a higher mutation rate, including TTN, MUC16, MET, MUC4, KMT2D, LRP2, and PCLO. This finding is consistent with previous analyses of gene mutation burden, implying that the lower-risk subgroup may be more responsive to immunotherapy. Following that, we chose medications presently used to treat cancer and assessed their susceptibility in various risk categories (Figure 9E–N). Interestingly, we discovered that patients with a high-risk score had lower IC50 values for the majority of drugs, including A-770041 (Lck targeted inhibitor), ABT-888 (small-molecule inhibitors of PARP, veliparib), AG-014699 (PARP inhibitors, rucaparib), AICAR (5-aminoimidazole-4-carboxamide ribonucleotide), and AMG-706 (a multikinase inhibitor, motesanib). Certain medications’ IC50 values were considerably lowered in individuals with low-risk scores, including AKT inhibitors and AS601245 (a selective JNK inhibitor). When these findings are combined, they imply that CRG is linked with medication sensitivity.

## Discussion

Although several targeted agents have recently been introduced in clinical applications for patients with high-grade RCC, the evidence for their efficacy in PRCC is not yet strong enough (Motzer et al., 2015; Choueiri and Kaelin, 2020). There are very few cases of PRCC, so the results of genetic tests and randomized control trials are often not included or only make up a very small part of the results of RCC (Courthod et al., 2015). Furthermore, since PRCC is distinct from clear cell RCC, the relevant study findings for clear cell RCC do not apply to PRCC (Massari et al., 2019). As a result, it is important to look into the molecular processes that cause these diseases and to find new biomarkers for targeted therapy. Tsvetkov et al. found that FDX1 and protein acylation (LIPT1, LIAS, DLDDLAT, PDHA1, and PDHB) were the main regulators of copper ionophore-induced cell death, and the knockout of seven genes prevented the killing of two copper ion carriers (Tsvetkov et al., 2022). We began by examining the gene mutation and expression of cuproptosis-related genes using data from the TCGA-PRCC and GSE2748 datasets. CNV-deficient CRGs, such as FDX1, DLAT, and DPT, were expressed at lower levels in PRCC samples than in normal renal samples, suggesting that CNV regulates CRG mRNA expression. As a result, the genomic and transcriptome landscape in CRGs is critical for controlling the onset and development of PRCC. CNV are somatic mutations in the DNA sequence that during the course of malignancy. The altered chromosomal structures are produced by an increase or decrease in the copy number of DNA segments, which is common in many types of cancer. In PRCC, somatic CNV identified three distinct tumor groupings. One grouping was often characterized by numerous gains of chromosomes 7p and 17p, deletion of the Y chromosome, and further gains; the majority of these cancers were type 1 and of low grade (Ren et al., 2018). Somatic mutations are non-heritable changes to the human genome that arise in somatic cells on their own accord (Futreal et al., 2004). Linehan et al. indicated that MET mutations are mostly found in type 1 cancers and in the tyrosine kinase domain.

Following that, we grouped PRCC patients according to their expression of cuproptosis-related genes, resulting in two unique pyroptotic patterns. Furthermore, evaluating the clinicopathological characteristics of different CRG subclusters revealed significant differences in CRG transcription and pathological stage. Additionally, we detected substantial changes in CRG expression across various cuproptosis patterns, with all CRGs being downregulated in CRG cluster B and upregulated in CRG cluster A. The GSVA analysis revealed that CRG cluster A was significantly enriched in tumor-associated pathways. According to our findings, the infiltration of most immune cells differed significantly between the two subclusters. Activated B cells, activated CD4 T cells, and natural killer T cells were infiltrated in much greater numbers in subcluster B than in subcluster A. Cellular metabolism-associated pathways were found to be significantly overrepresented by DEGs. Using a combined study of mutation and CNV, numerous pathways were identified as often dysregulated in PRCC. Wnt, Notch, TGF-, and Hedgehog signaling pathways were shown to be enhanced in type 1 PRCC (Saleeb et al., 2018). Additionally, when type 1 tumor tissue is compared to normal renal tissue, numerous intriguing pathways have been found, including adherens junction, focal adhesions, TGF signaling, Wnt signaling, and MAP kinase signaling. We conducted an unsupervised cluster analysis on the 739 DEGs associated with prognosis to group PRCC patients into three distinct gene subclusters. Patients with gene subcluster B had the poorest overall survival, while patients in gene subcluster A had the best OS. CRGs expression differed significantly amongst the three cuproptosis gene subclusters, as predicted based on the cuproptosis patterns. We computed risk scores across testing and training sets to confirm the risk score’s predictive performance. Survivability studies showed that patients in the lower-than-normal risk category had a considerably better prognosis. Mei et al. (2022) constructed a cuproptosis-related signature that was used to classify clear cell renal cell carcinoma patients into distinct risk clusters, with low-risk patients having a much better prognosis. Because the risk score is inconvenient to apply in practice, we created a nomogram that combines the risk score with clinicopathological variables to estimate patient survival time.

As demonstrated in the scatter graphs, the risk score was positively linked with B cells, M1 Macrophages, and CD4 memory activated T cells. We identified a substantial association between the majority of immune cells and three genes, including APEH and M0 macrophages, CLYBL and M2 macrophages, and ZNF844 and CD4 memory resting T cells. A greater risk score was also shown to be substantially associated with a higher immunological score, stromal score, and ESTIMATE score. Our examination of the mutation datasets revealed that the higher-risk category had a lower TMB than the lower-risk category, suggesting that the lower-risk category may benefit from immunotherapy. In recent years, renewed interest in immunotherapy has been sparked by the discovery that PD-1 and its ligand PD-L1 are expressed in the majority of RCC (Choueiri and Motzer, 2017). In addition to T- and B-cells, natural killer cells, and macrophages, the PD-1 receptor is found in other immune cells as well. Various malignancies cells may express it, even though it is seldom expressed in healthy cells (Johnson et al., 2018). One study indicated that after initiating therapy with nivolumab in multiple patients with advanced PRCC, computed tomography scans around half a year later revealed a considerable decrease in the size and quantity of systemic metastases (Adrianzen Herrera et al., 2017). Following that, we chose medications presently used to treat cancer and assessed their susceptibility in various risk categories. Interestingly, we discovered that patients with a high-risk score had lower IC50 values for the majority of drugs, including A-770041 (Lck targeted inh ibitor) and AMG-706 (a multikinase inhibitor, motesanib). Certain medications’ IC50 values were considerably lowered in individuals with low-risk scores, including AKT inhibitors and AS601245 (a selective JNK inhibitor). When these findings are combined, they imply that CRG is linked with medication sensitivity. In a phase II trial50, Foretinib, a dual MET/VEGFR2 inhibitor, was recently assessed in 74 participants with PRCC (Choueiri et al., 2013). Five out of ten (50%) of these participants had a RECIST partial response, whereas the remaining individuals achieved stable disease as their best response. There are no conventional medicines that have been shown to be effective in the treatment of metastatic PRCC. A clinical experiment at the National Cancer Institute is now evaluating one method that aims to exploit these cancers’ reliance on aerobic glycolysis and a high glucose flow (Chen et al., 2019).

Indeed, there is growing evidence that copper is a dynamic signaling molecule that exerts significant control over a varied array of activities, including lipolysis, cellular proliferation, autophagy, and brain activity (Tsang et al., 2020). Copper’s growing involvement in maintaining or restoring homeostasis emphasizes the critical nature of controlling its biological availability both within and outside the cell (Ackerman and Chang, 2018). It is thought that mutations in the ATP7A/B family, which are identical enzymes, cause the hereditary copper transport diseases Menkes and Wilson illness (Kaler, 2013). Genetic investigations have shown unequivocally that export is the primary mechanism of protection against copper toxicity, since cells lacking ATP7A are substantially more susceptible to excess copper than those lacking metallothioneins (Gudekar et al., 2020). Current antineoplastic drugs have significant off-target consequences because they often target fundamental characteristics of cells that are shared by all rapidly reproducing cells (Oliveri, 2022). The goal of developing new therapeutic medicines should be to improve selectivity and thereby minimize adverse effects. Additionally, these drugs should overcome resistance to tumor cells and specifically target tumor stem cells. There are some copper ionophores that have shown promise in this field because they are naturally good at causing cuproptosis in tumor cells instead of healthy ones. Disulfiram (DSF) and other copper ionophores have been looked at as antitumor drugs that can cause cuproptosis (Ge et al., 2022). It has been useful in treating alcoholism for over half a century as a commonly used aldehyde dehydrogenase inhibitor. Since it has various biological functions, it’s becoming more popular to repurpose DSF as an anticancer drug (Ekinci et al., 2019). DSF’s inexpensive cost, great availability, safety profile, and antitumor efficacy have piqued the curiosity of researchers (Kannappan et al., 2021). A number of cancer cell lines have shown DSF to be an antitumor drug in recent years (Li et al., 2020). Additionally, previous research has shown that co-administration of DSF with copper greatly enhances its antitumor activity since DSF’s active form is a copper complex of DTC. DSF’s toxic effects seem to be directly connected to the intracellular buildup of copper that DSF promotes (Cen et al., 2004). Despite DSF’s good outcomes *in vitro* and *in vivo*, clinical trials in malignancy sufferers were unsuccessful (Kannappan et al., 2021). This discouraging result might be explained by the quick degradation of DSF and its active component or by the use of a distinct route of administration for DSF and copper. It's worth mentioning that long-term use of copper-binding drugs, such as copper ionophores, might disrupt vital metal homeostasis, resulting in significant adverse effects in individuals undergoing the medication. Whereas copper ionophores have demonstrated inherent selectivity against tumor cells, as stated above, their therapeutic window has to be expanded for safer use. As a result, current research has concentrated on establishing logical methodologies and innovative therapeutic modalities to improve tumor cell targeting.

## Conclusion

To summarize, the cuproptosis-related gene signature is important for the definition of the TME and the predication of PRCC prognosis. The risk score of a single tumor may help us better understand the peculiarities of TME invasion and aid in the development of more effective immunotherapy tactics.

## Data Availability

The original contributions presented in the study are included in the article/supplementary material, further inquiries can be directed to the corresponding author.
